# Paclitaxel-induced painful neuropathy is associated with changes in mitochondrial bioenergetics, glycolysis, and an energy deficit in dorsal root ganglia neurons

**DOI:** 10.1097/j.pain.0000000000000939

**Published:** 2017-05-02

**Authors:** Natalie A. Duggett, Lisa A. Griffiths, Sarah J.L. Flatters

**Affiliations:** Wolfson Centre for Age-Related Diseases, Institute of Psychiatry, Psychology and Neuroscience, King's College London, London, United Kingdom

**Keywords:** Chemotherapy-induced neuropathy, Neurotoxicity, Sensory neuron, Mitochondria, Taxol, Dorsal root ganglia

## Abstract

Painful neuropathy is the major dose-limiting side effect of paclitaxel chemotherapy. Mitochondrial dysfunction and adenosine triphosphate (ATP) deficit have previously been shown in peripheral nerves of paclitaxel-treated rats, but the effects of paclitaxel in the dorsal root ganglia (DRGs) have not been explored. The aim of this study was to determine the bioenergetic status of DRG neurons following paclitaxel exposure in vitro and in vivo. Utilising isolated DRG neurons, we measured respiratory function under basal conditions and at maximal capacity, glycolytic function, and Adenosine diphosphate (ADP)/ATP levels at 3 key behavioural timepoints; prior to pain onset (day 7), peak pain severity and pain resolution. At day 7, maximal respiration and spare reserve capacity were significantly decreased in DRG neurons from paclitaxel-treated rats. This was accompanied by decreased basal ATP levels and unaltered ADP levels. At peak pain severity, respiratory function was unaltered, yet glycolytic function was significantly increased. Reduced ATP and unaltered ADP levels were also observed at the peak pain timepoint. All these effects in DRG neurons had dissipated by the pain resolution timepoint. None of these paclitaxel-evoked changes could be replicated from in vitro paclitaxel exposure to naive DRG neurons, demonstrating the impact of in vivo exposure and the importance of in vivo models. These data demonstrate the nature of mitochondrial dysfunction evoked by in vivo paclitaxel in the DRG for the first time. Furthermore, we have identified paclitaxel-evoked changes in the bioenergetics of DRG neurons, which result in a persistent energy deficit that is causal to the development and maintenance of paclitaxel-induced pain.

## Abstract 2

Supplemental Digital Content is Available in the Text.

Investigation into the main energy producing pathways prior to, during, and at the resolution of paclitaxel-induced pain in the cell bodies of sensory neurons.

## 1. Introduction

Paclitaxel is a first-line taxane-derived chemotherapeutic agent commonly used in the treatment of breast and ovarian cancers. The clinical use of paclitaxel is impeded by peripheral neuropathy, which remains the main dose-limiting side effect because of the current lack of preventative or treatment approaches (reviewed in Ref. 16). Patients typically report numbness, tingling, spontaneous pain, and evoked pain to mechanical and cold stimuli in their hands and feet.^3,5,23^ Recent meta-analysis showed that paclitaxel-induced peripheral neuropathy affected 44% to 98% of patients.^25^ Furthermore, neuropathy can persist for months or years after the paclitaxel treatment ends.^5,25,28^ Thus, paclitaxel treatment is a significant cause of sensory abnormalities and chronic pain substantially affecting quality of life.^27^

Several rodent models of paclitaxel-induced painful neuropathy have been developed (reviewed in Ref. 17). We have used a rat model evoked by low doses of clinically formulated paclitaxel, which mimics the scenario of multiple nonconsecutive systemic injections (akin to treatment cycles), evoking mechanical/cold hypersensitivities, with similar time-courses to those seen in patients.^11,12,15^ Previous studies with this model found an increased frequency of atypical (swollen and vacuolated) mitochondria in C-fibres and myelinated axons of the saphenous (purely sensory) nerve prior to and during paclitaxel-induced pain, but not at its resolution.^12^ In addition, decreased complex I and II-derived respiration and adenosine triphosphate (ATP) levels were found in an isolated sciatic (sensory and motor) nerve preparation prior to and during paclitaxel-induced pain.^31^ Recently, we have linked paclitaxel-induced mitochondrial dysfunction to oxidative stress in vivo. We have shown that inhibition of complex III, a mitochondrial reactive oxygen species (ROS) producing site, evoked antinociceptive effects on the development and maintenance of paclitaxel-induced pain.^15^ Furthermore, we found evidence that paclitaxel induces neuronal-derived mitochondrial ROS in the saphenous nerve, dorsal root ganglion (DRG), and spinal cord, which is poorly controlled by endogenous antioxidant systems.^8^

Swollen mitochondria were also observed in the DRG of paclitaxel-treated rats,^2^ suggesting paclitaxel affects mitochondrial function within the cell bodies of sensory axons as well as at mid-axon level. Mitochondria are essential to the cell; governing ATP production, calcium buffering, ROS production, and a number of key signalling pathways. However, given the multiple functions of mitochondria, morphological changes cannot define the nature of mitochondrial dysfunction. In addition, both oxidative phosphorylation and glycolysis produce ATP. Thus, to fully understand the bioenergetic status combined analysis of oxidative phosphorylation, glycolysis and adenine nucleotide content is required. Therefore, the aim of this study is to determine the bioenergetic status of DRG neurons following paclitaxel exposure in vitro and in vivo. Specifically, utilising isolated DRG neurons, we have measured respiratory function under basal conditions and at maximal capacity, glycolytic function, and adenosine diphosphate (ADP) and ATP levels. These data give novel mechanistic insight into the bioenergetic environment in sensory neuronal cell bodies prior to, during, and at the resolution of paclitaxel-induced mechanical hypersensitivity, providing significant understanding of paclitaxel-induced mitochondrial dysfunction and energy production in relation to pain behaviour. Data from these studies were previously presented in abstract form.^7^

## 2. Methods

### 2.1. Animals

Adult male Sprague-Dawley rats (starting weight 180-220 g; Harlan, UK) were kept in a climate controlled room containing only rats. Rats were housed in groups of 3 to 4 in plastic cages with sawdust bedding and environmental enrichment materials. Bedding/cages were changed twice a week. Artificial light was provided on a 12-hour light-dark cycle (lights on at 7 am) and standard food and water were freely available. Throughout all studies, rats were routinely visually checked and weighed to ensure good health. Health status prior to treatment was normal. All studies were carried out in accordance with the UK Animals (Scientific Procedures) Act, 1986 and the IASP ethical guidelines.^32^ Protocols were approved by the Ethical Review Panel of King's College London and conducted under the UK Home Office project licenses 70/6673 & 70/8015.

### 2.2. Administration of paclitaxel

The clinical formulation of 6 mg/mL Paclitaxel Solution for Infusion (CP Pharmaceuticals Ltd, UK or Actavis Ltd, UK) was diluted with 0.9% sterile saline (Fresenius Kabi, United Kingdom) to achieve a 2 mg/mL solution. To replicate the clinical formulation of paclitaxel, a vehicle stock solution was made consisting of equal parts of cremophor EL (Sigma, Dorset, United Kingdom) and ethanol. In experiments using paclitaxel solution manufactured by Actavis Ltd, the vehicle stock solution also contained 2 mg/mL sodium citrate as per this formulation. For vehicle administration; 1 part vehicle stock solution was diluted with 2 parts 0.9% sterile saline. Rats received intraperitoneal 2 mg/kg paclitaxel or equivalent volume of vehicle solution on 4 alternate days (0, 2, 4, and 6). Animals were dosed according to their weight, ie, 250 g rat received 0.25 mL injection. Injections were performed in the morning, and rats were immediately returned to their home cages afterwards.

### 2.3. Behavioural assessment of mechanical hypersensitivity

**Figure 1.**
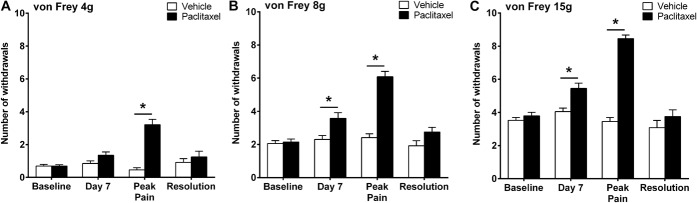
Time course of paclitaxel-induced mechanical hypersensitivity. Graphs show the mean ± SEM of the number of withdrawal responses to (A) 4 g, (B) 8 g, and (C) 15 g von Frey filaments at baseline, day 7, peak pain (day 24-44), and pain resolution (day 170-218), following paclitaxel or vehicle administration at days 0, 2, 4, and 6. *P < 0.05, 2-tailed multiple comparison unpaired *t* tests with Holm-Sidak correction. Baseline & day 7 n = 40 vehicle, n = 40 paclitaxel; peak pain n = 24 vehicle, n = 24 paclitaxel; resolution n = 12 vehicle, n = 12 paclitaxel.

As previously described,^11,15^ rats were habituated to the testing environment, and mechanical hypersensitivity was assessed by withdrawal responses to von Frey filaments with bending forces of 4, 8, and 15 g. Three baseline measurements were taken prior to paclitaxel or vehicle administration and mechanical hypersensitivity was measured at 1 to 3 week intervals until the paclitaxel-induced pain syndrome had resolved. Overall, paclitaxel-induced mechanical hypersensitivity has a delayed onset, reaching maximal hypersensitivity by day 24, remaining at the maximal plateau for several weeks and eventually resolves from day 170 onwards. Three critical timepoints of paclitaxel-induced mechanical hypersensitivity were investigated in these studies (Fig. 1): day 7—24 hours after the last injection of paclitaxel, prior to emergence of mechanical hypersensitivity; day 24 to 44—peak of mechanical hypersensitivity (von Frey responses recorded as ≥2.2 fold higher than baseline responses); day 170 to 218—resolution of mechanical hypersensitivity (return to individual baseline responses observed on 2 separate occasions). Variability in the timepoint of harvest at peak pain and resolution of pain was dictated by the pain behaviour displayed by individual rats. The total number of rats used at each timepoint was as follows; day 7—16 vehicle, 16 paclitaxel; peak pain—12 vehicle, 12 paclitaxel; resolution of pain—12 vehicle, 12 paclitaxel. Sample sizes were based on the number of animals used in a given experiment.

### 2.4. Isolation of dorsal root ganglion neurons

Rats were overdosed with pentobarbital, and DRGs were dissected bilaterally starting from L6/L5 proceeding rostrally and placed in warmed Minimum Essential Media (MEM; Sigma) containing 1% penicillin/streptomycin (P/S; Invitrogen, Paisley, United Kingdom). Fourteen DRGs were harvested per animal. Further dissection removed the ventral root, and partially removed the dura mater and dorsal root. Dorsal root ganglia were transferred to fresh MEM containing 1% penicillin/streptomycin and 2.5 mg/mL collagenase (type IV; Worthington Biochemicals, United Kingdom) and incubated at 37°C for 3 hours. Dorsal root ganglia were triturated and trypsin (0.25 mg/mL; Sigma) added for 10 to 20 minutes at 37°C. The single-cell suspension was then diluted with MEM containing 10% fetal bovine serum (FBS; Sigma) 1% P/S, and centrifuged at 1000 rpm for 5 minutes. The pellet was resuspended in MEM containing 1% P/S, 10% FBS, and 0.1 mg/mL DNase I (Worthington Biochemicals, Reading, United Kingdom) and pipetted onto a 15% bovine serum albumin (BSA), (w/v in MEM; Sigma) cushion and centrifuged for 10 minutes at 1000 rpm. The layer of debris and myelin formed at the solution interface was discarded before removing BSA cushion and media to leave a cell pellet. This pellet was resuspended in MEM containing 1% P/S, 10% FBS, and 0.1% cytosine arabinoside (Sigma) before plating. The cell suspension was plated directly onto XF24 Analyzer multi-well plates (for bioenergetic profile analysis); white 96-well plates (for ADP/ATP analysis); clear 96-well plates and poly-d-lysine–coated size 0 glass coverslips (for cell counts). All plates were incubated overnight at 37°C, 5% CO_2_, before assessment of the bioenergetic profile or ADP/ATP levels, the following day.

### 2.5. XF24 Extracellular Flux Analyser and standardization of assays

The bioenergetic profile refers to the measurement of respiration and glycolysis under basal conditions and at maximal capacity. The XF24 Extracellular Flux Analyzer (Seahorse Bioscience, Billerica, MA) enables the simultaneous measurement of extracellular flux changes in oxygen and protons in media immediately surrounding adherent cells in a multiwell plate format—for more details see Refs. 10, 22—providing readouts in terms of oxygen consumption rate (OCR) and extracellular acidification rate (ECAR). Four injection ports (A-D) in the cartridge, above the multiwell plate containing cells, allow the bioenergetic response of cells to different drugs or compounds which specifically modulate mitochondrial function, to be quantified.^10,22^ Extensive preliminary experiments identified the ideal standard conditions for these experiments by determining the optimal cell density, XF24 measurement parameters and concentrations of compounds which modulate mitochondrial function. Timings of mix-wait-measure XF24 cycles allowed ample re-oxygenation and pH equilibration between measurements (see Supplementary Figure 1, available online at http://links.lww.com/PAIN/A412). Several carbonyl cyanide p-(trifluoromethoxy) phenylhydrazone (FCCP) (Sigma) titration experiments (0.05-0.8 μM) were conducted to determine the highest concentration (0.2 μM) that maximally increased respiration without causing the proton motive force to collapse (see Supplementary Figure 2, available online at http://links.lww.com/PAIN/A412). In addition, oligomycin titration experiments (0.5-1.5 μM) were also performed and found no difference in the degree of response (see Supplementary Figure 3, available online at http://links.lww.com/PAIN/A412).

### 2.6. Normalisation of bioenergetic profiles

After completion of all experiments, DRG cells were fixed in cold 4% PFA for 15 minutes, washed with PBS, and nuclei stained with DAPI (1:5000 in PBS) at room temperature, in the dark. XF24 multiwell plates were then scanned on an INCell Analyzer 100 (GE Healthcare Life Sciences, Amersham, United Kingdom). Twenty-five images (positioned between well standoffs) were taken over the area from which OCR and ECAR measurements were recorded. The individual images were then processed in CellProfiler software, using a freely available DAPI pipeline, (version 2.1.1; Broad Institute, Cambridge, MA) to count the total number of cells, to which OCR/ECAR measurements were then normalised. Nonmitochondrial respiration was subtracted from basal respiration, ATP turnover, proton leak, and maximal respiration values (detailed in Ref. 4). As expected, the enzymatic dissociation of DRG to isolate DRG neurons resulted in the presence of nonneuronal cells. The contribution of nonneuronal cells to the bioenergetic profile was therefore investigated. Because of high adherence of nonneuronal cells to plasticware compared with neurons, wells were washed repeatedly with MEM shortly after plating to preferentially remove the majority of neurons. Oxygen consumption rate/ECAR measurements on predominantly nonneuronal cells identified nonneuronal cells as minimal contributors to the bioenergetic profiles observed when DRG neurons were present (see Supplementary Figure 4, available online at http://links.lww.com/PAIN/A412). In addition, samples of DRG cell suspensions from paclitaxel- and vehicle-treated rats were plated on poly-d-lysine–coated glass coverslips, fixed with cold 4% PFA and stained with DAPI and Neu-N. Cell counts identified the proportion of neuronal and nonneuronal cells. Dorsal root ganglion cell preparations contained 8% to 12% neurons. The proportion of neurons did not significantly change between treatment groups at any of the 3 timepoints.

### 2.7. Assessment of bioenergetic profiles of dorsal root ganglion neurons

**Figure 2.**
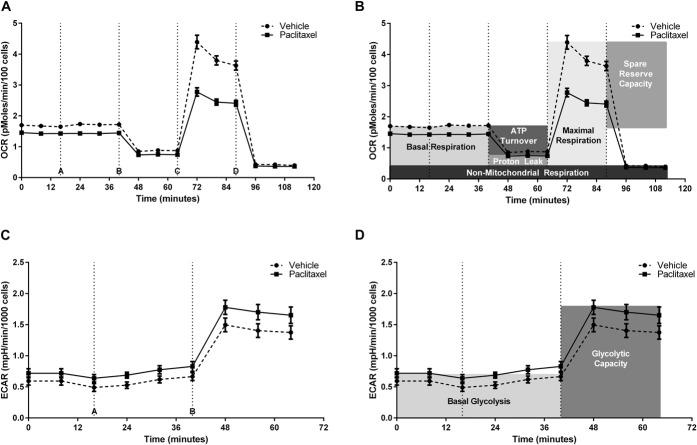
Measurement of bioenergetic profiles of isolated dorsal root ganglion (DRG) neurons using the Seahorse XF24 analyzer. (A and B) show the OCR of isolated DRG neurons from paclitaxel-/vehicle-treated animals at day 7, under basal conditions and at maximal capacity, normalised to cell number. Data are expressed as mean ± SEM of 7 wells of cells from one paclitaxel- or vehicle-treated rat. Dashed lines indicate injection of media + DMSO (A; control), oligomycin (B), FCCP (C), antimycin A and rotenone (D). (B) shows the same graph as (A), with the 6 different parameters of respiratory function shaded. (C and D) show the ECAR of isolated DRG neurons from paclitaxel-/vehicle-treated animals at peak pain severity, under basal conditions and at maximal capacity, normalised to cell number. Data are expressed as mean ± SEM of 8 wells of cells from 1 paclitaxel- or vehicle-treated rat. Dashed lines indicate injection of media + DMSO (A; control), oligomycin (B). (D) shows the same graph as (C), with the 2 parameters of glycolytic function shaded. ATP, adenosine triphosphate; OCR, oxygen consumption rate; ECAR, extracellular acidification rate.

On the day of the experiment, XF Assay unbuffered media (Seahorse Bioscience) containing 2 mM GlutaMAX was warmed to 37°C, supplemented with 5 mM glucose (Sigma), 1 mM sodium pyruvate (Fisher Scientific, Loughborough, United Kingdom), and pH adjusted to pH 7.4. Cells were washed in this media, and the multiwell plate was placed into a 37°C, 0% CO_2_ incubator for approximately 45 minutes. Compounds which specifically modulate mitochondrial function were used to determine the bioenergetic profile: oligomycin—inhibits ATP synthase; FCCP—ionophore that uncouples ATP synthesis from electron transport chain function; rotenone—inhibits complex I; antimycin A—inhibits complex III (all from Sigma). All compounds were dissolved in DMSO (dimethyl sulfoxide; Fisher) to 2.5-mM stock solutions. These stock solutions were diluted with freshly prepared XF media (described above) and loaded into injection ports of the cartridge. During experiments, 3 OCR/ECAR measurements were recorded prior to and following each addition from injection ports (A-D). Following these additions, DRG cells were sequentially exposed to compounds in the following order: (A) 0.4% DMSO—as control for maximal DMSO concentration of subsequently injected reagents; (B) 0.5 μM or 1 μM oligomycin; (C) 0.2 μM FCCP; (D) 1 μM antimycin A and 1 μM rotenone (Fig. 2A). Six aspects of respiratory function are assessed because of the effects of these compounds on OCR: basal respiration; oxygen consumption attributable to with ATP turnover; proton leak; maximal respiration; spare reserve capacity (the respiratory ability of the cell to respond to/overcome stress); and nonmitochondrial respiration (illustrated in Fig. 2B). Extracellular acidification rate measurements were simultaneously assessed from the same DRG preparations following 0.4% DMSO and oligomycin additions (Fig. 2C). Two aspects of glycolytic function were then determined: basal glycolysis and glycolytic capacity—the tendency of cells to switch to glycolysis from oxidative phosphorylation (illustrated in Fig. 2D).

Following in vivo behavioural assessment, DRG cells from 1 paclitaxel-treated rat and 1 concurrent vehicle-treated rat were harvested (as described above) at each timepoint of interest. Bioenergetic profiles were determined for each animal from 5 to 10 wells of DRG cells. Sample sizes per treatment group: day 7, n = 6 animals; peak pain (day 24-29), n = 5 animals; resolution of pain (day 170-191), n = 5 animals. Bioenergetic profiles of naive DRG cells were also determined following in vitro paclitaxel exposure prior to or during OCR/ECAR measurements to assess the effect of preexposure and the presence of acute paclitaxel on oxygen consumption and glycolysis. Naive DRG cells were exposed for 1 hour to either paclitaxel (10 μM or 10 nM; Sigma, in XF assay media solution containing 1% DMSO—paclitaxel initially dissolved in DMSO), vehicle (XF assay media containing 1% DMSO) or XF assay media alone at 37°C, 5% CO_2_. Cells were then washed with XF assay media and bioenergetic profiles determined as described above. In separate experiments, OCR/ECAR measurements were taken for 1 hour after naive isolated DRG cells were exposed to paclitaxel (10 μM or 10 nM in XF assay media solution containing 1% DMSO), vehicle (XF assay media containing 1% DMSO), or XF assay media alone following baseline OCR/ECAR measurements to determine immediate effects on OCR/ECAR. Cells were then washed with XF assay media and bioenergetic profiles determined as described above. In both sets of experiments, the 4 experimental groups—control, vehicle, 10 μM paclitaxel, 10 nM paclitaxel were run in parallel in the same multiwell plates. Bioenergetic profiles of naive DRG cells following acute paclitaxel exposure were determined from 2 to 3 wells per animal, n = 5 animals. Paclitaxel concentrations used in these experiments were based on reported plasma concentrations in humans following paclitaxel infusion.^14^

### 2.8. Assessment of adenosine diphosphate/adenosine triphosphate levels in dorsal root ganglion neurons

Six wells containing 50-μL aliquots of DRG cell suspensions were plated in white-walled (for ADP/ATP analysis) and clear 96 well plates (for cell counts) per animal and kept overnight at 37°C, 5% CO_2_. ADP/ATP Assays (ab65313; Abcam, United Kingdom) were used per kit instructions. Background luminescence was measured following the addition of 100 μL of reaction mix (1:9, ATP monitoring enzyme: nucleotide releasing buffer [NRB]) to empty wells. Media (MEM) was removed from wells containing DRG cells and 50 μL of NRB added. Cells were incubated for 2 minutes at RT. 100 μL of reaction mix was then added to wells containing cells, and the luminescence recorded (ATP level). Subsequently, 1 µL of ADP-converting enzyme was added, incubated for 2 minutes, and luminescence recorded again (ADP level). Adenosine triphosphate standards were measured concurrently, and concentrations of ATP and ADP in cell replicates interpolated from a standard curve. Adenosine triphosphate standards were made from a 1 mg stock of ATP (Sigma) reconstituted in NRB. Luminescence values were normalised to cell numbers taken from concurrent clear-walled 96 well plates, fixed in cold 4% PFA for 15 minutes and nuclei stained with DAPI (1:5000 in PBS). Plates were scanned on an INCell Analyzer 100, taking 34 images of the bottom of the wells. Cell counts were then determined using CellProfiler software. Following in vivo behavioural assessment, DRG cells from 1 paclitaxel-treated rat and 1 concurrent vehicle-treated rat were harvested (as described above) at each timepoint of interest. Adenosine diphosphate/ATP levels were measured as described above from 6 to 8 wells per animal. Sample sizes per treatment group: day 7, n = 10 to 11 animals; peak pain (day 28-44), n = 7 animals; resolution of pain (day 182-218), n = 7 animals. In separate experiments, naive DRG cells were exposed for 1 hour to either paclitaxel (10 μM or 10 nM; Sigma, in media containing 1% DMSO—paclitaxel initially dissolved in DMSO), vehicle (media containing 1% DMSO) or media alone at 37°C, 5% CO_2_, to determine the effect of in vitro paclitaxel on ADP/ATP levels. Adenosine diphosphate/ATP levels was measured from 5 wells of DRG cells per condition from each naive animal, n = 4 animals.

### 2.9. Statistics

All statistical analyses were conducted using GraphPad Prism 6 or GraphPad InStat 3 for Windows. To assess development of paclitaxel-induced mechanical hypersensitivity, 2-tailed multiple-comparison unpaired *t* tests with Holm-Sidak correction compared paclitaxel-treated rats to timepoint-matched vehicle-treated rats. Changes in OCR and ECAR measures between paclitaxel- and vehicle-treated rats were analysed using 2-tailed paired *t* tests. This was to enable direct pairwise comparison between DRG neurons from each harvest on a given day (consisting of 1 paclitaxel-treated rat and 1 vehicle-treated rat) which were then measured in parallel in the same multiwell plate. Given the observed changes in these measures, 1-tailed unpaired *t* tests were then used to compare differences in ADP/ATP levels of DRG neurons from paclitaxel- and vehicle-treated rats. Effects of in vitro paclitaxel or vehicle exposure on OCR and ECAR measures, and ADP/ATP levels in naive DRG neurons were analysed using 1-way ANOVA with Dunnett post hoc analysis comparing to control group. Statistical significance was accepted at *P* < 0.05. No further distinction has been made when *P* < 0.01 or *P* < 0.001 and is denoted on the figures as *P* < 0.05.

## 3. Results

We have consistently found that 4 systemic low-dose (2 mg/kg) injections of paclitaxel administered on days 0, 2, 4, and 6, evokes mechanical hypersensitivity with a gradual onset. Paclitaxel-induced mechanical hypersensitivity takes several weeks to reach its peak, remains elevated for a few months, and eventually resolves in approximately 6 months following the first paclitaxel injection. In these studies, we examined isolated DRG neurons at 3 key timepoints within the time course of paclitaxel-induced mechanical hypersensitivity; (1) day 7—24 hours after the last injection of paclitaxel, prior to paclitaxel-induced pain behaviour, (2) day 24 to 44—peak of paclitaxel-induced pain behaviour and (3) day 170 to 218—resolution of paclitaxel-induced pain behaviour. Figure 1 shows the combined behavioural data from several paclitaxel- and vehicle-treated cohorts, demonstrating the pain phenotype evident before tissue isolation for these investigations. As typically expected, we observed a ≥2.2-fold significant increase in paw withdrawal responses to von Frey 4, 8, and 15 g stimulation at the peak of paclitaxel-induced mechanical hypersensitivity (Fig. 1, *P < 0.05, 2-tailed multiple comparison unpaired *t* tests with Holm-Sidak correction, n = 12-40 animals). In individual cohorts of animals, we did not observe significant differences in mechanical hypersensitivity between paclitaxel-treated rats and vehicle-treated rats at day 7. However, when these data were collated, small but statistically significant increases in responses to von Frey 8 and 15 g at day 7 (Fig. 1B, C) were seen in paclitaxel-treated rats (*P < 0.05, 2-tailed multiple comparison unpaired *t* tests with Holm-Sidak correction). It is highly likely that the statistical significance evident at these timepoints is due to the high n numbers, n = 40 at day 7, following data collation from multiple cohorts rather than a biological effect.

The XF24 Extracellular Flux Analyser used here enables analysis of mitochondrial function from intact cells without permeabilisation or isolating mitochondria. We avoided these techniques because dysfunctional, swollen mitochondria would not survive the necessary technical processes, and therefore create a bias to normal mitochondria, which is not representative of the scenario in vivo. Not all mitochondrial substrates and inhibitors eg, succinate can permeate the cell membrane, therefore examination of complex II-mediated respiration in isolated DRG neurons was not possible. Preliminary experiments identified the ideal standard experimental conditions for assessing bioenergetic profiles of DRG neurons (see Supplementary Figures 1-3, available online at http://links.lww.com/PAIN/A412). Figure 2 illustrates typical bioenergetic profile of DRG neurons isolated from paclitaxel- and vehicle-treated rats and subsequent measures of respiratory function at day 7 (Fig. 2A, B) and glycolytic function at peak pain (Fig. 2C, D). We determined that nonneuronal cells had a negligible impact on the bioenergetic profile both in terms of respiratory and glycolytic function (Supplementary Figure 4, available online at http://links.lww.com/PAIN/A412).

**Figure 3.**
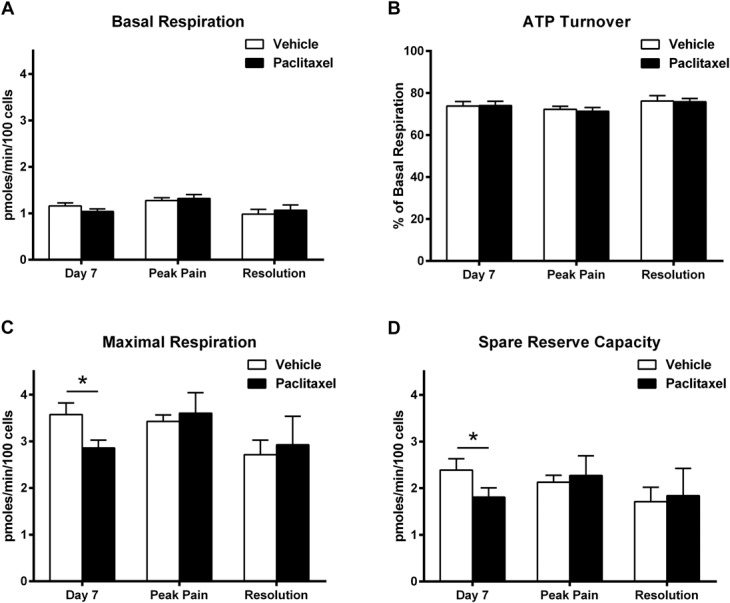
Respiratory function of dorsal root ganglion (DRG) neurons in paclitaxel- and vehicle-treated rats during the time course of paclitaxel-induced pain. Graphs show the mean ± SEM of (A) basal respiration, (B) oxygen consumption associated with ATP turnover-linked respiration, (C) maximal respiration, and (D) spare reserve capacity of DRG neurons isolated from paclitaxel- and vehicle-treated rats at day 7, peak pain and resolution of pain timepoints, n = 5 to 6 animals, per treatment group, per timepoint (5-10 replicates from each animal). *P < 0.05 paired 2-tailed *t* tests.

Figure 3 illustrates the changes in respiratory function of isolated DRG neurons of paclitaxel-treated rats compared to vehicle-treated rats, prior to, during, and at the resolution of paclitaxel-induced pain. There was no significant difference in the basal respiration or ATP turnover-linked respiration at any timepoint investigated (Fig. 3A, B). However, there was a significant reduction in the maximal respiration and spare reserve capacity of DRG neurons from paclitaxel-treated rats at day 7 compared to DRG neurons from concurrent vehicle-treated rats (Fig. 3C, D, *P < 0.05, paired 2-tailed *t* tests). This indicates that paclitaxel impairs the ability of mitochondria to increase their respiratory function under conditions of stress or higher energy demand. These effects on mitochondrial respiratory function were not observed at peak pain or pain resolution timepoints.

**Figure 4.**
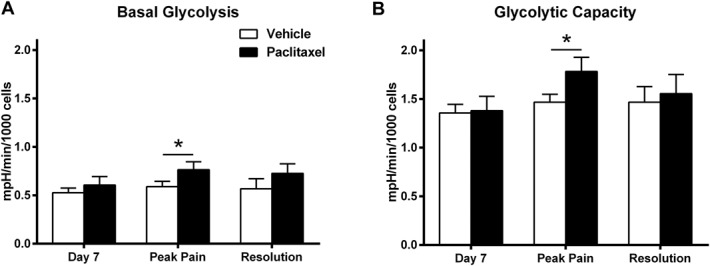
Glycolytic function of dorsal root ganglion (DRG) neurons in paclitaxel- and vehicle-treated rats during the time course of paclitaxel-induced pain. Graphs show the mean ± SEM of (A) basal glycolysis and (B) glycolytic capacity of DRG neurons isolated from paclitaxel- and vehicle-treated rats at day 7, peak pain and resolution of pain timepoints, n = 5 to 6 animals, per treatment group, per timepoint (5-10 replicates from each animal). *P < 0.05 paired 2-tailed *t* tests.

In conjunction with investigating the oxygen consumption rate (OCR), extracellular acidification rate (ECAR) was concurrently measured, relaying information regarding the glycolytic function of the isolated DRG neurons prior to, during, and at the resolution of paclitaxel-induced pain. In contrast to the changes in oxygen consumption observed at day 7, basal glycolysis and glycolytic capacity remained unchanged at this timepoint (Fig. 4A, B). However, at the peak pain timepoint, basal glycolysis and glycolytic capacity were significantly increased in DRG neurons isolated from paclitaxel-treated rats compared to DRG neurons from concurrent vehicle-treated rats (Fig. 4A, B, *P < 0.05, paired 2-tailed *t* tests). Examining DRG neurons in XF assay media without glucose confirmed that higher basal glycolysis was associated with the peak of paclitaxel-induced pain (paclitaxel 1.520 ± 0.098 mpH/min per 1000 cells; vehicle: 1.207 ± 0.042 mpH/min per 1000 cells, *P* = 0.005, 2-tailed paired *t* test). At the resolution of paclitaxel-induced pain, there was no significant difference between paclitaxel- and vehicle-treated rats in these glycolytic measures. The proportion of neurons vs nonneuronal cells in isolated DRG preparations from paclitaxel- and vehicle-treated rats did not significantly change at any timepoint investigated. Thus, our observed changes in paclitaxel-treated rats are not due to changes in nonneuronal cell function given that their presence did not vary between groups and they also have a negligible contribution to the bioenergetic profile (Supplementary Figure 4, available online at http://links.lww.com/PAIN/A412).

**Figure 5.**
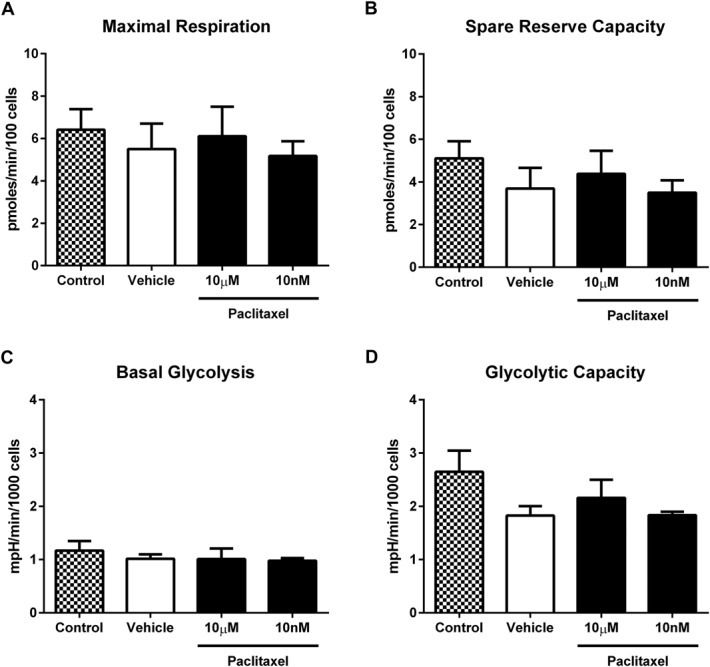
Respiratory and glycolytic function of naive dorsal root ganglion (DRG) neurons during in vitro paclitaxel exposure. Graphs show the mean ± SEM of (A) maximal respiration, (B) spare reserve capacity, (C) basal glycolysis, and (D) glycolytic capacity of naive DRG neurons during exposure to paclitaxel (10 μM or 10 nM), vehicle (1% DMSO), or control (media only).

Given that paclitaxel has been previously shown to remain within the DRG at day 7 following this dosing regimen,^30^ we investigated if in vitro paclitaxel exposure to naive isolated DRG neurons affected respiratory or glycolytic function. Bioenergetic profiles of naive DRG neurons were assessed either in the direct presence of paclitaxel or following prior preexposure to paclitaxel. One-hour exposure to 10 nM or 10 μM paclitaxel had no effect on basal respiration or ATP turnover-linked respiration (data not shown). In addition, the presence of paclitaxel (10 nM or 10 μM) had no effect on maximal respiration, spare reserve capacity, basal glycolysis, or glycolytic capacity (Fig. 5A, D). Similarly, 1-hour exposure to paclitaxel (10 nM or 10 μM) prior to assessment of bioenergetic profiles also had no effect on basal respiration, ATP turnover-linked respiration, maximal respiration, spare reserve capacity, basal glycolysis, or glycolytic capacity (data not shown).

**Figure 6.**
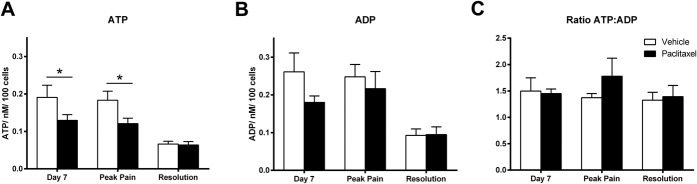
Levels of adenosine triphosphate (ATP), adenosine diphosphate (ADP), and the ratio of ATP:ADP in dorsal root ganglion (DRG) neurons from paclitaxel- and vehicle-treated rats during the time course of paclitaxel-induced pain. Graphs show the mean ± SEM of (A) ATP levels and (B) ADP levels of DRG neurons isolated from paclitaxel- and vehicle-treated rats at day 7, peak pain and resolution of pain timepoints. (C) shows the mean ± SEM of the ratio of ATP:ADP from the same DRG neuronal preparations. n = 7 to 11 animals, per treatment group, per timepoint (6-8 replicates from each animal). *P < 0.05 1-tailed unpaired *t* tests.

**Figure 7.**
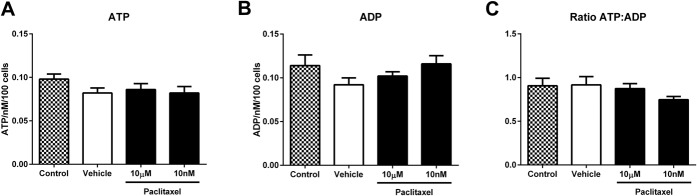
Levels of adenosine triphosphate (ATP), adenosine diphosphate (ADP), and the ratio of ATP:ADP in naive dorsal root ganglion (DRG) neurons following in vitro paclitaxel exposure. Graphs show the mean ± SEM of the concentration of (A) ATP levels and (B) ADP levels of naive DRG neurons following exposure to paclitaxel (10 μM or 10 nM), vehicle (1% DMSO), or control (media only). (C) shows the mean ± SEM of the ratio of ATP:ADP from the same naive DRG neuronal preparations. n = 4 animals, with 5 replicates from each animal, per condition.

Adenosine triphosphate synthase is the only complex of the mitochondrial electron transport chain that can work bidirectionally to respond to the energy needs of the cell either converting ADP to ATP or vice versa. Thus, measuring only ATP levels in cells may not fully demonstrate the nature of mitochondrial dysfunction (discussed further in Ref. 4) and energy production status. Therefore, given our observed changes in mitochondrial and glycolytic function, we measured ADP and ATP levels of DRG neurons isolated from paclitaxel and vehicle-treated rats, prior to, during, and at the resolution of paclitaxel-induced pain behaviour (Fig. 6). Adenosine triphosphate levels in DRG neurons from paclitaxel-treated rats were significantly decreased at day 7 and peak pain timepoints compared to DRG neurons from concurrent vehicle-treated rats (Fig. 6A, *P < 0.05 1-tailed unpaired *t* tests). Similar ATP levels of DRG neurons from paclitaxel- and vehicle-treated rats were observed at pain resolution. There was no statistically significant change in ADP levels of DRG neurons from paclitaxel-treated rats compared to DRG neurons from vehicle-treated rats at day 7, peak pain or pain resolution timepoints (Fig. 6B). The ratio of ATP:ADP did not significantly alter in DRG neurons from paclitaxel-treated rats at any of the 3 timepoints investigated. The effects of in vitro paclitaxel exposure on ADP/ATP levels in naive DRG neurons were also examined (Fig. 7). One-hour exposure to paclitaxel (10 nM or 10 μM) or vehicle (1% DMSO) had no effect on ATP or ADP levels, or in the ratio of ATP:ADP compared to control neurons (Fig. 7).

## 4. Discussion

Bioenergetic pathways and mitochondrial electron transport activity are tightly controlled to enable cell survival with spontaneous adaptation to maintain cellular homeostasis. Therefore, any change in the capacity of these pathways can have significant impact on cellular function. For the first time, we have examined oxidative phosphorylation, glycolysis, and ADP/ATP levels to understand how the main energy producing pathways alter prior to, during, and at the resolution of paclitaxel-induced pain in DRG neurons.

At day 7, 24 hours after the last paclitaxel administration and prior to marked pain behaviour, both maximal respiration and spare reserve capacity were significantly impaired in DRG neurons isolated from paclitaxel-treated rats. This suggests that although basal respiration is unaffected, the ability of DRG neurons to respond to stress or higher energetic demand is diminished by paclitaxel. Paclitaxel is still present within the DRG at day 7 in this rat model.^30^ However, direct in vitro paclitaxel exposure either prior to or during assessment of respiratory function had no significant effect on respiratory function under basal conditions or at maximal capacity. This suggests that prolonged in vivo exposure of paclitaxel is required for impairment of mitochondrial function in DRG neurons. Significantly less ATP was found in DRG neurons from paclitaxel-treated rats at day 7 compared to concurrent vehicle controls under normal conditions. These effects could not be replicated in naive DRG neurons following in vitro paclitaxel exposure, further demonstrating the importance of in vivo modelling. The decrease in ATP levels was not accompanied by a significant increase in ADP levels suggesting that ATP synthase functionality is not reversed. Furthermore, paclitaxel did not affect ATP turnover-linked respiration suggesting that the machinery and process of ATP generation is not impaired by paclitaxel directly. Dorsal root ganglion neurons from paclitaxel-treated rats at day 7 do not preferentially switch to glycolysis from oxidative phosphorylation, thus lower ATP levels are not due to a paclitaxel-evoked enhanced glycolytic function. Therefore, we suggest that the deficit in ATP levels observed at day 7 could be due to enhanced release or leak of ATP from DRG neurons.

At the peak of paclitaxel-induced mechanical hypersensitivity, measures of respiratory function under basal conditions or at maximal capacity were unaltered in DRG neurons from paclitaxel-treated rats compared to concurrent vehicle controls, suggesting a normalisation of mitochondrial function. However, the glycolytic function of DRG neurons from paclitaxel-treated rats was significantly increased at peak pain. This was evident in terms of basal glycolytic function, but also glycolytic capacity ie, an increased tendency to switch to glycolysis. This preference for glycolysis occurred despite unlimited substrates for oxidative respiration. We also observed significantly decreased levels of ATP in DRG neurons from paclitaxel-treated rats at peak pain severity. As glycolysis is less efficient than oxidative phosphorylation at producing ATP, reduced ATP levels at peak pain may well be a result of this paclitaxel-evoked enhancement of glycolytic function. We did not observe any significant change in respiratory function, glycolytic function, or ADP/ATP levels at the pain resolution timepoint. This further indicates that changes in the bioenergetic status and ATP levels in DRG neurons observed at day 7 and peak pain timepoints are contributory factors to the development and maintenance of paclitaxel-induced pain.

Previous experiments measured oxygen consumption and ATP levels from an ex vivo teased sciatic nerve preparation in the same paclitaxel rat model used here at day 7 and peak pain timepoints only.^31^ Despite different methodological approaches, comparison between our data using DRG neurons and data with sciatic nerves^31^ provides further understanding of causal mechanisms of paclitaxel-induced painful neuropathy. Prior to paclitaxel-induced pain, maximal respiration is impaired in both DRG neurons and sciatic nerves, but basal respiration at both sites is unaltered. At the peak of paclitaxel-induced pain, basal respiration is unaffected in both DRG neurons and sciatic nerves, yet impaired maximal respiration is only seen in sciatic nerves. The effects of paclitaxel on ATP levels are the same, prior to, and during paclitaxel-induced pain. Less ATP was present in DRG neurons in situ, yet deficits in ATP production in sciatic nerves are only observed during maximally stimulated conditions. Collectively, this could suggest that mitochondrial dysfunction and ATP deficit in both DRG neurons and peripheral nerves initiates the development of paclitaxel-induced pain. Additionally, it is possible that mitochondrial dysfunction continues to be factor in the maintenance of paclitaxel-induced pain in peripheral nerves specifically, whereas mitochondrial function recovers in DRG neurons, yet an energy deficit persists because of the preferential switch to glycolysis.

A switch to glycolysis may act as a part of a protective mechanism for DRG neurons exposed to paclitaxel in vivo. The dosing regimen of paclitaxel utilised in this study does not increase ATF3 staining in DRG,^12^ despite observation of swollen, abnormal mitochondria in the DRG.^2^ We have also observed increased ROS levels in IB4+ DRG neurons and spinal neurons prior to pain onset (day 7).^8^ Several reports link ROS, and reactive nitrogen species (RNS), to the initiation of apoptosis (reviewed in Ref. 21); however, this is not evident in this low-dose paclitaxel model. Neurons extensively metabolise glucose.^24^ Glucose metabolism via the pentose-phosphate pathway (PPP) generates reduced NADPH, which maintains glutathione levels. Maintained glutathione levels keep cytochrome C in an inactive state and thus prevent initiation of apoptosis. These pathways have been shown to inhibit apoptosis in DRG neurons.^29^ The PPP and glycolysis are interlinked (reviewed in Ref. 20). This suggests that the increased basal glycolysis and glycolytic capacity observed here in DRG neurons from paclitaxel-treated rats at the peak pain timepoint may be indicative of increased PPP activity and thus glutathione levels. Considering that the endogenous antioxidant enzyme GPx utilises glutathione as a substrate (to convert hydrogen peroxide to water), increased glutathione may account for the increased GPx activity we observed in DRG from paclitaxel-treated rats at the peak pain timepoint.^8^ The enhanced glycolytic function of DRG neurons from paclitaxel-treated rats observed here at peak pain could perhaps be an adaptive mechanism to produce less ROS.

In recent years, ROS and RNS have been shown to be causal factors in paclitaxel-induced pain through behavioural studies with pharmacological scavenging agents.^6,11,13,18,19^ Mitochondria are a substantial source of ROS. Our previous study showed elevated mitochondrial ROS in DRG and spinal neurons prior to development of paclitaxel-induced pain.^8^ The consequences of this elevated ROS are likely potentiated by a delayed endogenous antioxidant response.^8^ Furthermore, we have shown that inhibition of complex III, a mitochondrial ROS producing site, inhibited the development and maintenance of paclitaxel-induced pain.^15^ Collectively, these data suggest that ROS/RNS are significant drivers in the paclitaxel-induced pain syndrome. Aside from ROS production, mitochondria are also involved in other critical cellular pathways that could have a substantial role in chemotherapy-induced pain. Here, we have shown compromised bioenergetic function and decreased ATP levels in DRG neurons prior to development of paclitaxel-induced pain. This suggests that it is unlikely a single chain of events occurs to evoke pain following chemotherapy. Alternatively, there are multiple factors connected to mitochondria that may interlink or occur simultaneously to initiate and potentially maintain paclitaxel-induced pain. It is to be determined precisely how paclitaxel-induced mitochondrial dysfunction translates to behavioural hypersensitivity. Given the importance of ATP to the maintenance of pump activity which regulates ion exchange, eg, Na^+^/K^+^ ATPase, it is conceivable that ATP deficit could have consequences on ion exchange in axons potentially driving aberrant electrical activity. It has been estimated that ∼50% of the ATP in neurons is utilised for the maintenance of ion exchange—discussed in Refs. 1, 9. Another possibility is presynaptic Ca^2+^ clearance mechanisms, as electrical stimulation of capsaicin-sensitive DRG neurons showed that presynaptic Ca^2+^ clearance was predominantly dependent upon Ca^2+^-ATPase and mitochondria.^26^

In summary, we have examined how the main energy producing pathways alter prior to, during, and at the resolution of paclitaxel-induced pain in the cell bodies of sensory neurons. In vivo paclitaxel acutely evokes deficits in mitochondrial bioenergetics in DRG neurons, prior to the emergence of pain behaviour, which is accompanied by decreased ATP levels. During paclitaxel-induced pain, DRG neurons preferentially switch to glycolysis potentially explaining the sustained decrease in ATP levels. These data elucidate the nature of mitochondrial dysfunction evoked by paclitaxel in vivo and highlight a persistent energy deficit in DRG neurons. These events are likely important factors in the development and maintenance of paclitaxel-induced painful neuropathy. This study provides further evidence of differential mechanisms occurring at the initiation compared to the maintenance of paclitaxel-evoked pain syndrome. Identifying these mechanisms and their temporal importance will aid a targeted approach to the development of drugs which can prevent or reverse chemotherapy-induced painful neuropathy.

## Conflict of interest statement

The authors have no conflicts of interest to declare.

N. A. Duggett was supported by The Wellcome Trust (WT093335AIA). 2010 round 2 Research Grant from The Royal Society awarded to S. J.L. Flatters contributed to the purchase of the Seahorse XF24 Extracellular Analyser used in these studies. S. J.L. Flatters, L. A. Griffiths and part of these studies were supported by a Capacity Building Award in Integrative Mammalian Biology funded by the BBSRC, BPS Integrative Pharmacology Fund, HEFCE, DIUS, MRC and SFC. The Wellcome Trust (WT093335AIA) also funded these studies.

## Supplementary Material

SUPPLEMENTARY MATERIAL

## Appendix A. Supplemental Digital Content

Supplemental Digital Content associated with this article can be found online at http://links.lww.com/PAIN/A412.
