# Supplementary figures and images for: Paclitaxel-induced painful neuropathy is associated with changes in mitochondrial bioenergetics, glycolysis, and an energy deficit in dorsal root ganglia neurons
Source: Pain. 2017 May 2;158(8):1499–508. doi: 10.1097/j.pain.0000000000000939 (PMC5515641; doi:10.1097/j.pain.0000000000000939)

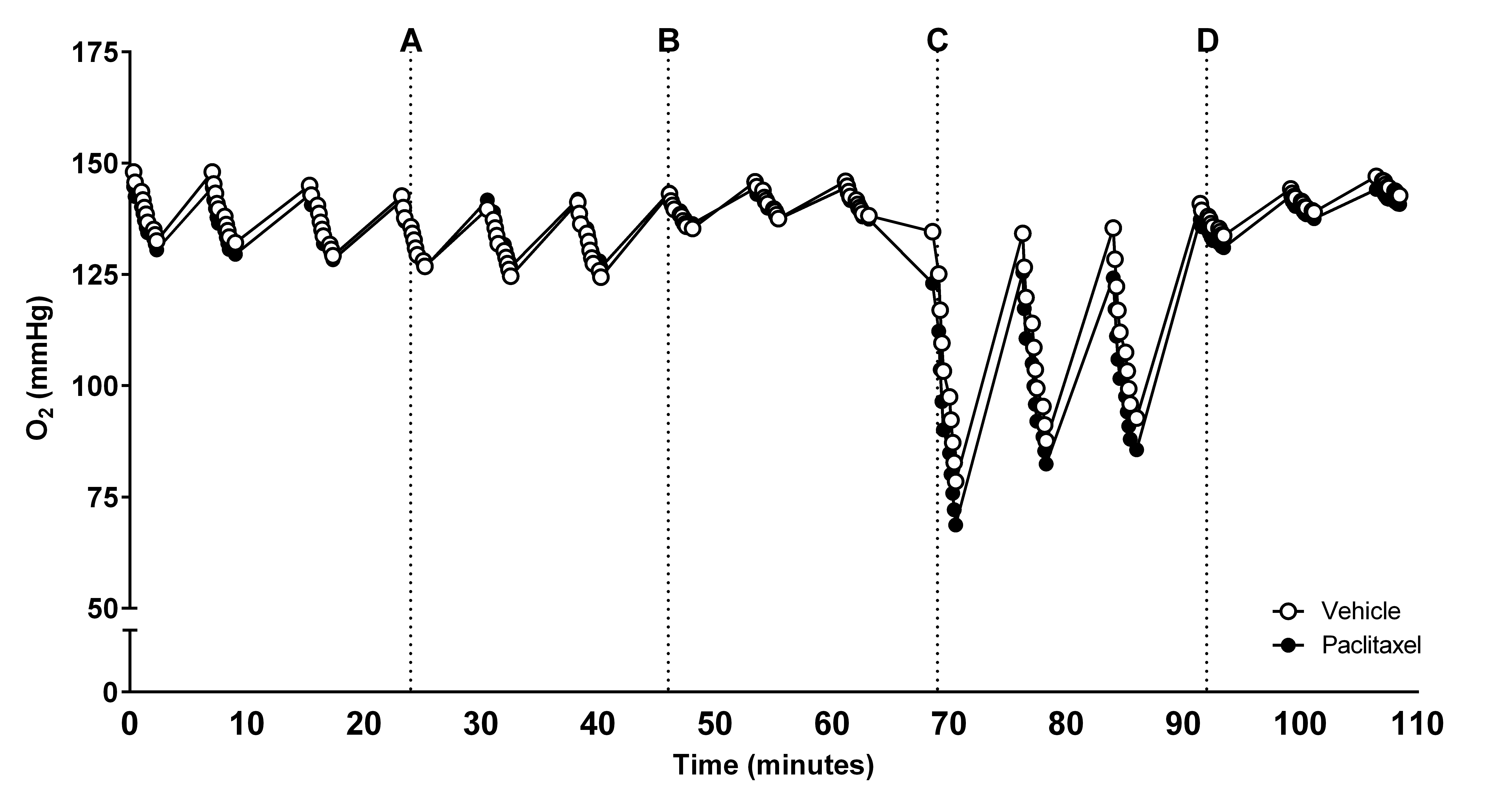

Supplement: SUPPLEMENTARY MATERIAL [file jop-158-1499-s001.tif]

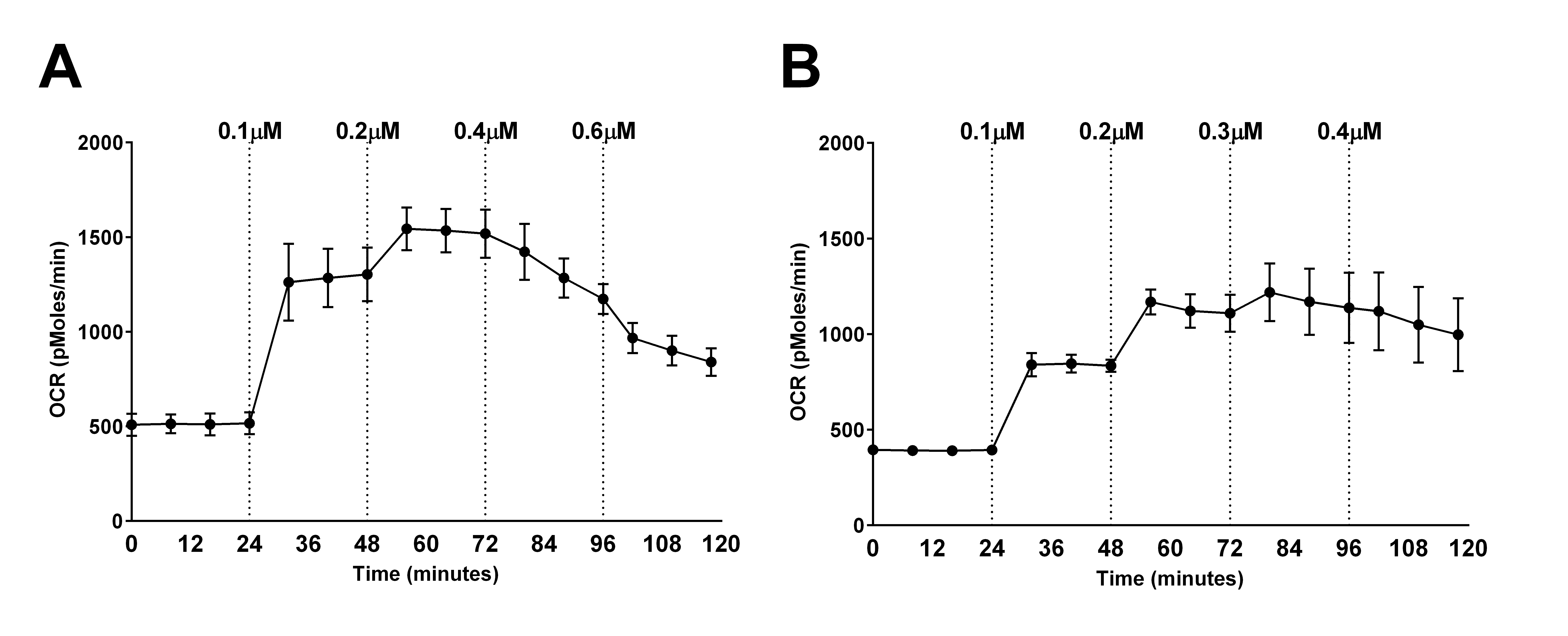

Supplement: SUPPLEMENTARY MATERIAL [file jop-158-1499-s002.tif]

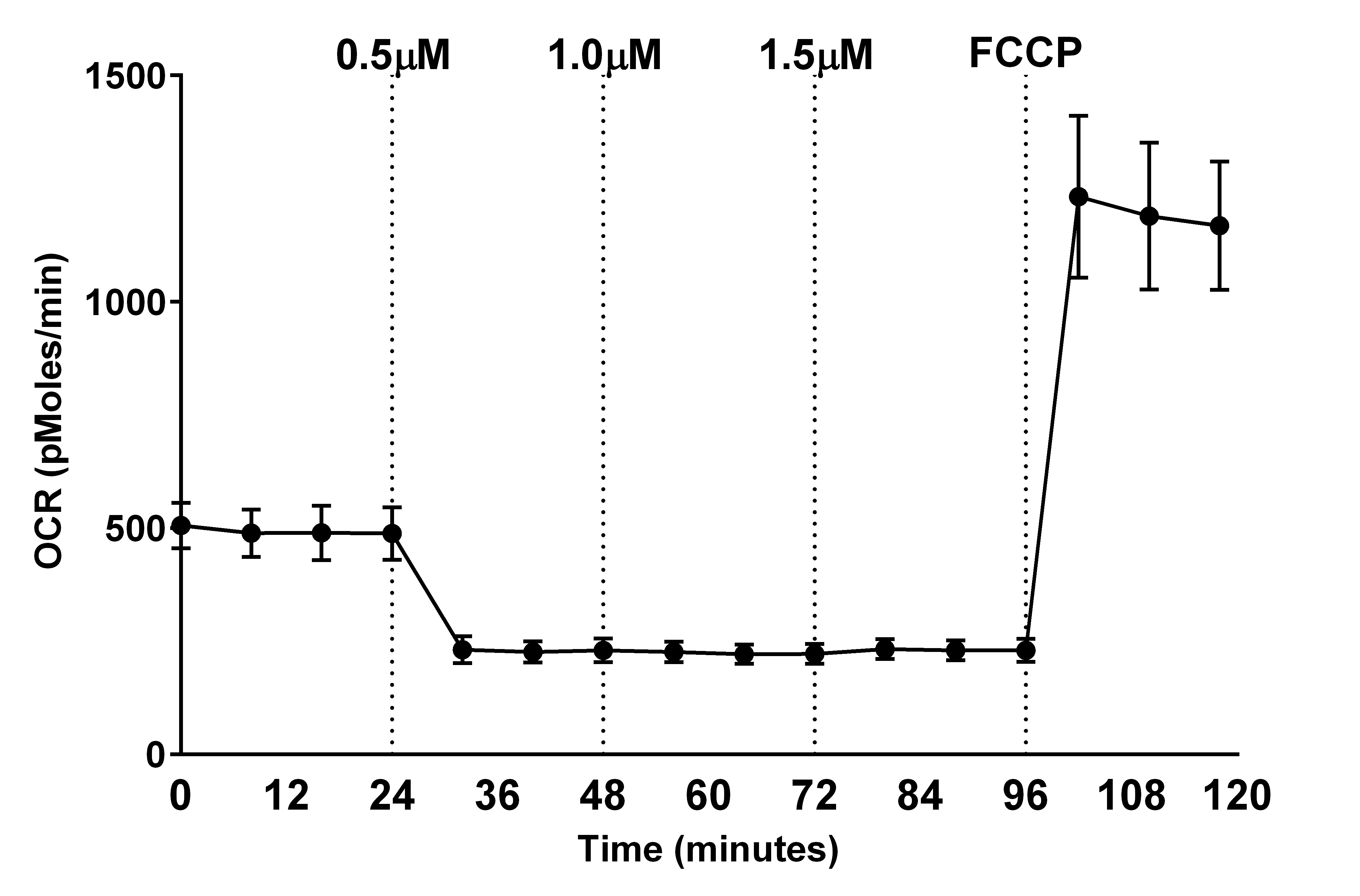

Supplement: SUPPLEMENTARY MATERIAL [file jop-158-1499-s003.tif]

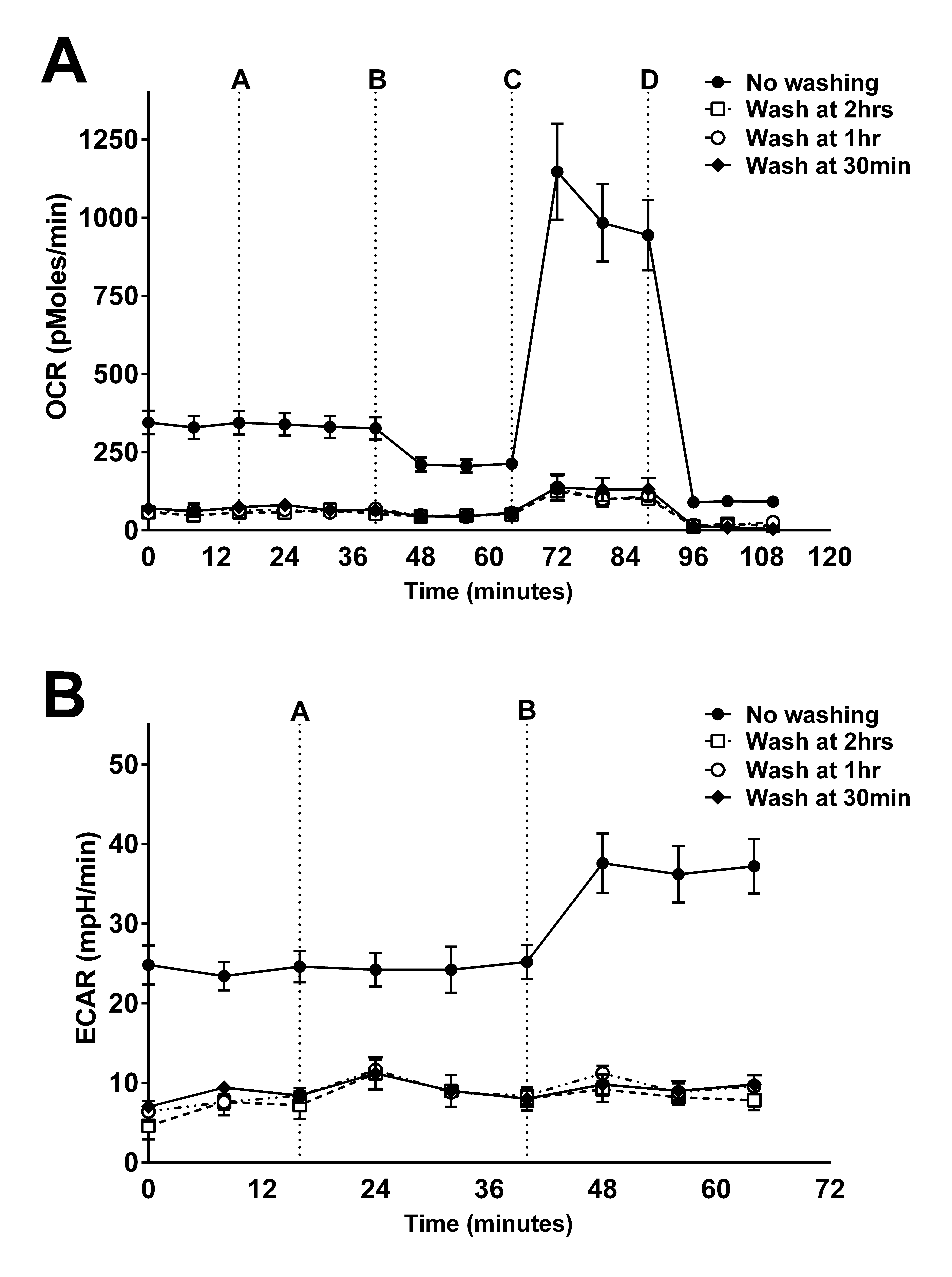

Supplement: SUPPLEMENTARY MATERIAL [file jop-158-1499-s004.tif]
